# Recent Advancement in Chitosan-Based Nanoparticles for Improved Oral Bioavailability and Bioactivity of Phytochemicals: Challenges and Perspectives

**DOI:** 10.3390/polym13224036

**Published:** 2021-11-22

**Authors:** Syed Sarim Imam, Sultan Alshehri, Mohammed M. Ghoneim, Ameeduzzafar Zafar, Omar Awad Alsaidan, Nabil K. Alruwaili, Sadaf Jamal Gilani, Md. Rizwanullah

**Affiliations:** 1 Department of Pharmaceutics, College of Pharmacy, King Saud University, Riyadh 11451, Saudi Arabia; salshehri1@ksu.edu.sa; 2 Department of Pharmacy Practice, College of Pharmacy, AlMaarefa University, Ad Diriyah 13713, Saudi Arabia; mghoneim@mcst.edu.sa; 3 Department of Pharmaceutics, College of Pharmacy, Jouf University, Sakaka 72341, Saudi Arabia; azafar@ju.edu.sa (A.Z.); osaidan@ju.edu.sa (O.A.A.); Nkalruwaili@ju.edu.sa (N.K.A.); 4 Department of Basic Health Sciences, Preparatory Year, Princess Nourah Bint Abdurrahman University, Riyadh 11671, Saudi Arabia; SJGlani@pnu.edu.sa; 5 Department of Pharmaceutics, School of Pharmaceutical Education and Research, Jamia Hamdard, New Delhi 110062, India; mdrizwanullah54@gmail.com

**Keywords:** phytochemicals, chitosan nanoparticles, mucoadhesion, bioavailability, bioactivity

## Abstract

The excellent therapeutic potential of a variety of phytochemicals in different diseases has been proven by extensive studies throughout history. However, most phytochemicals are characterized by a high molecular weight, poor aqueous solubility, limited gastrointestinal permeability, extensive pre-systemic metabolism, and poor stability in the harsh gastrointestinal milieu. Therefore, loading of these phytochemicals in biodegradable and biocompatible nanoparticles (NPs) might be an effective approach to improve their bioactivity. Different nanocarrier systems have been developed in recent decades to deliver phytochemicals. Among them, NPs based on chitosan (CS) (CS-NPs), a mucoadhesive, non-toxic, and biodegradable polysaccharide, are considered the best nanoplatform for the oral delivery of phytochemicals. This review highlights the oral delivery of natural products, i.e., phytochemicals, encapsulated in NPs prepared from a natural polymer, i.e., CS, for improved bioavailability and bioactivity. The unique properties of CS for oral delivery such as its mucoadhesiveness, non-toxicity, excellent stability in the harsh environment of the GIT, good solubility in slightly acidic and alkaline conditions, and ability to enhance intestinal permeability are discussed first, and then the outcomes of various phytochemical-loaded CS-NPs after oral administration are discussed in detail. Furthermore, different challenges associated with the oral delivery of phytochemicals with CS-NPs and future directions are also discussed.

## 1. Introduction

Phytonutrients/phytochemicals are the secondary metabolites of plants with complex chemical structures widely present in a variety of foods, vegetables, and herbal extracts [1,2]. Phytochemicals are generally not essential for the growth of plants, but they are advantageous for human health and used in the prevention and treatment of different ailments [1,2,3]. Pure phytochemicals or plant extracts of medicinal plants produce health benefits by various direct mechanisms such as scavenging free radicals, protease inhibition, metal chelation, and microtubule inhibition [4,5]. After oral administration, phytochemicals undergo the digestion process from the mouth to the intestine followed by absorption from the gastrointestinal tract (GIT) by different absorption mechanisms, eventually reaching the systemic circulation. After that, they are metabolized, and the degraded product is excreted via biliary/renal/pulmonary pathways [4,5,6,7]. The final concentration of phytochemicals in the systemic circulation (i.e., oral bioavailability of phytochemicals) exerts the actual in vivo bioactivity. Therefore, attaining the maximum oral bioavailability of phytochemicals is the major considerable factor in achieving excellent therapeutic efficacy/bioactivity. Extensive investigation has revealed that the oral administration of phytochemicals shows excellent therapeutic efficacy in the management of a variety of diseases.

Apart from the excellent therapeutic potential of phytochemicals, the oral bioavailability and bioactivity of these phytochemicals are restricted due to their high lipophilicity, limited aqueous solubility, poor stability in the harsh gastrointestinal (GI) milieu, and limited intestinal absorption [8,9]. The limited aqueous solubility is the most common reason behind the poor oral bioavailability of phytochemicals because they must be passed through the unstirred water layer present on the epithelium surface to reach the systemic circulation. Therefore, there is an utmost need to develop a delivery system for phytochemicals that can improve their aqueous solubility, stability in the GI milieu, and intestinal absorption in order to improve oral bioavailability; thereby, the bioactivity of phytochemicals can be utilized.

In recent decades, nanoparticle-based systems have gained increased attention for addressing the challenges related to phytochemicals for oral delivery [10]. A variety of NP-based systems have been developed and utilized to improve the physicochemical characteristics of phytochemicals. These nano-sized systems provide a wide range of advantages as they improve the aqueous solubility, colloidal stability, controlled release, intestinal absorption, and, thereby, oral bioavailability of encapsulated phytochemicals [11]. Among the various NP-based systems, chitosan (CS)-based NPs (CS-NPs) have been proved to be the most effective nanocarriers to enhance the oral bioavailability and bioactivity of a wide range of phytochemicals. Phytochemical-loaded CS-NPs show great advantages such as enhancement of aqueous solubility, stability in the GI milieu by protecting phytochemicals from different metabolizing enzymes and different pH conditions, slow and controlled release of phytochemicals, and enhanced intestinal permeation by improving GI retention time due to mucoadhesive properties, thereby improving oral bioavailability as well as bioactivity. Furthermore, due to the excellent mucoadhesive properties and small-sized particles, CS-NPs can easily be passed through the biological barriers, deliver the drug to the target site, and reduce the dose-related toxicity [12,13].

**Figure 1:**
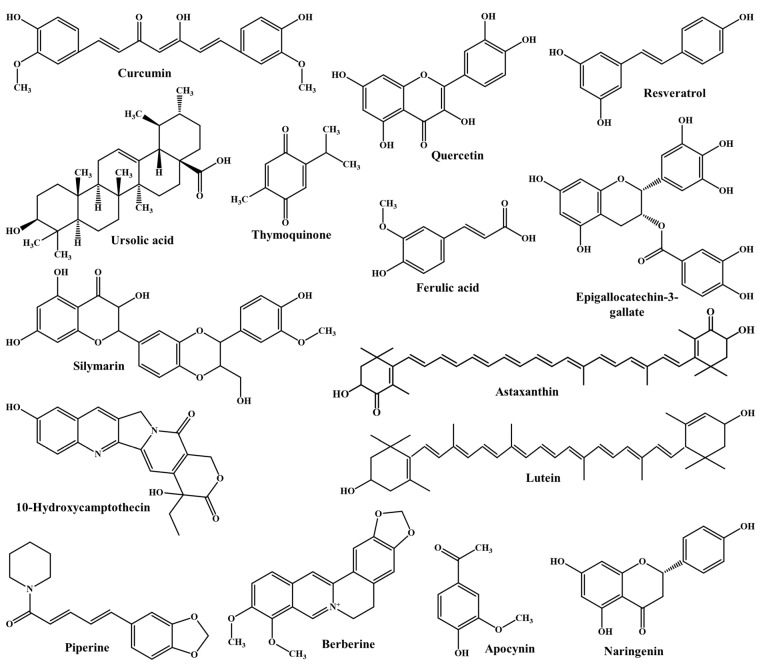
Chemical structures of selected phytochemicals reviewed in this manuscript for oral bioavailability and bioactivity after encapsulation in CS-NPs.

In this review, we particularly focus on phytochemical delivery via CS-NPs for improved oral bioavailability and bioactivity. The rationale behind the delivery of phytochemicals via CS-NPs and their mechanism of intestinal absorption are also discussed. After an extensive literature review, we selected 15 phytochemicals, discussing their CS-NP systems in particular. The chemical structures of the selected phytochemicals are depicted in Figure 1.

## 2. Physicochemical Properties of Phytochemicals and Challenges in Oral Delivery

Since ancient times, phytochemicals have gained immense importance worldwide due to their beneficial effects on human health and various diseases such as their antioxidant, anticancer, antidiabetic, anti-hypertensive, anti-hyperlipidemic, antimicrobial, antiviral, anti-inflammatory, and hepatoprotective effects [14,15]. Most phytochemicals are bioactive and are secondary metabolites of plants. Extensive studies have shown that these phytochemicals prevent and/or cure a variety of ailments by improvement in physical or mental performance. However, many scientists across the world have questioned the health benefit claims of phytochemicals due to the challenges associated with achieving actual therapeutic efficacy in humans [16,17]. As we all know, pure synthetic/semi-synthetic drugs have to be taken in a specific dose at a fixed time. However, phytochemicals are generally taken at low and variable levels as part of a normal diet at irregular intervals [18]. Consequently, it is very difficult to ascertain the specific health benefits after the consumption of phytochemicals. Moreover, the oral bioavailability and bioactivity of phytochemicals can vary after processing, long-term storage, and oral intake due to modulation in the physicochemical properties [18,19]. In addition, the bioavailability and bioactivity of phytochemicals are greatly affected by quantity, composition, and other foods consumed with particular phytochemicals [20,21]. Furthermore, phytochemicals greatly vary from each other in terms of polarity, charge, functional groups, and molecular weights, which is also a reason for the significant differences in their solubilities in different solvents, partitioning, stability in different environmental conditions, and physical states [8].

After oral administration of phytochemicals, firstly, they need to be solubilized in the gastrointestinal fluids (GIF). As discussed earlier, most phytochemicals have very limited solubility. Therefore, their low solubility in the GIF is one of the major challenges encountered during oral phytochemical delivery. Further, GIF show variation in pH from the stomach (pH 1.2) to the small intestine (pH 6.8), and the GIT has many metabolizing enzymes. Hence, most phytochemicals after oral administration are greatly degraded by the acidic pH of the GIT and metabolized due to metabolizing enzymes, resulting in the poor oral bioavailability of these drugs. Furthermore, the highly lipophilic characteristics of phytochemicals are also a major reason for their poor intestinal absorption and, thereby, low oral bioavailability. Altogether, high lipophilicity, poor stability in the GIF, and poor intestinal absorption are the most important reasons behind the poor oral bioavailability of phytochemicals [22,23]. Physicochemical characteristics of the selected phytochemicals reviewed in this manuscript are summarized in Table 1.

## 3. Chitosan: Source and Structure

**Figure 2:**
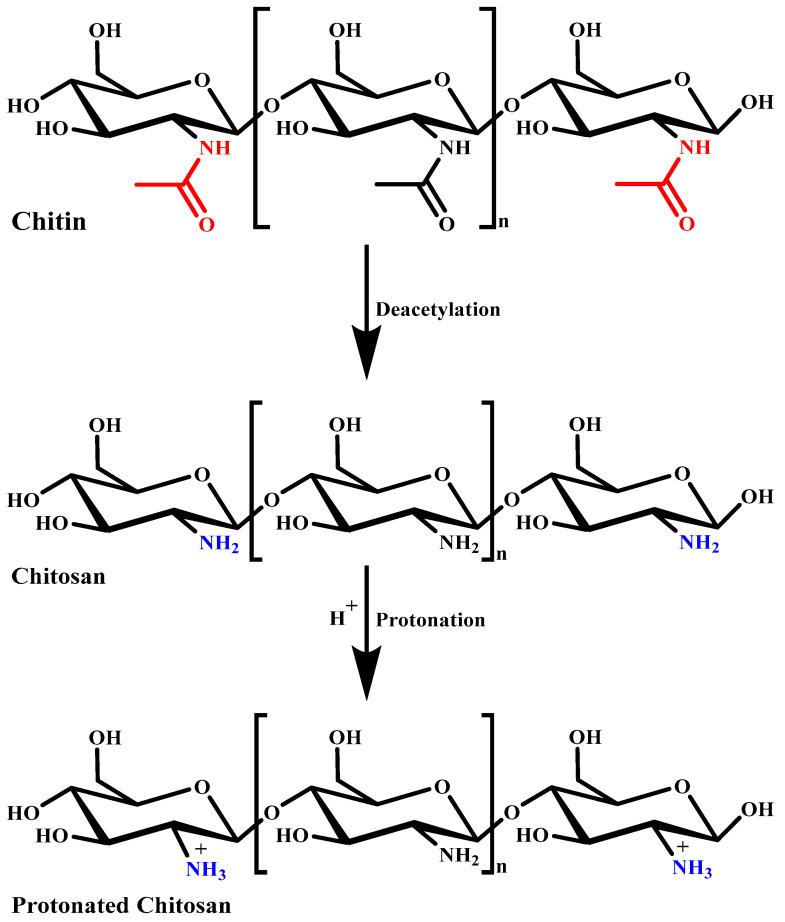
Chemical structure of chitin, chitosan, and protonated chitosan.

**Figure 3:**
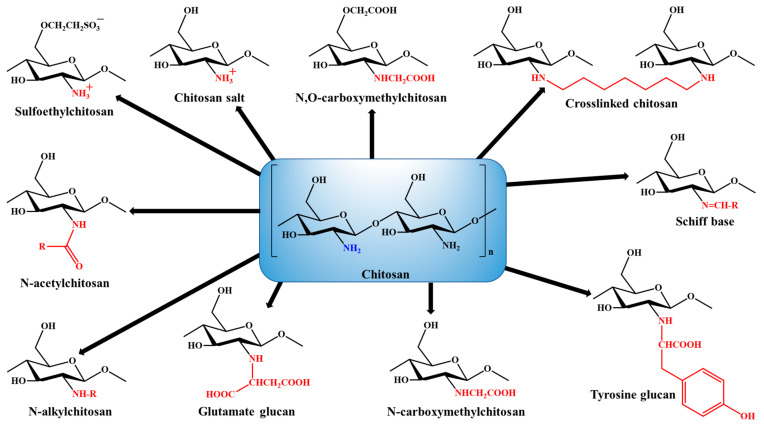
Chemical structures of different chitosan derivatives prepared by the functionalization of native chitosan.

In the last two decades, chitosan (CS) has become the most attractive polymer due to its unique physicochemical characteristics and wide range of applications in the healthcare system. CS is a natural mucopolysaccharide that has a very similar chemical structure to cellulose, with one acetylamino functional group instead of a hydroxyl group present at the C-2 position. Pure CS itself exhibits different biological properties, as summarized in Table 2 [39,40,41,42,43,44,45,46,47,48]. For commercial production, CS is manufactured by the alkaline N-deacetylation of chitin. Chitin is a naturally abundant biopolymer mostly obtained from the exoskeleton of crabs and shrimps [49]. The degree of acetylation greatly affects the physicochemical characteristics of CS. The degree of deacetylation of CS is particularly determined by the protonation of glucosamine and N-acetylglucosamine units [50]. Its physicochemical characteristics such as solubility in different solvents and pH conditions, toxicity, and hydrophobicity are highly dependent on its deacetylation degree and molecular weight, depending on the chitin source [51,52]. Figure 2 presents the process of chitin deacetylation and protonation of CS. The amino functional group present at the C-2 position of the glucosamine unit greatly strengthens the structural and functional characteristics of CS. The cationic characteristics of CS due to the amino group provide mucoadhesiveness and make it an excellent carrier for oral drug/phytochemical delivery. Its unique properties such as being biodegradable, biocompatible, and non-toxic, and having stability in diverse environmental and pH conditions make CS the most researched polymer for biomedical applications. The physicochemical characteristics of CS can be modified very easily by chemical or enzymatic functionalization approaches. Functionalization of the amino and hydroxyl groups of CS provides a wide range of functionalized N-modified, O-modified, and N,O-modified CS derivatives that show enhanced biological activity [53,54]. The chemical structures of different functionalized CS derivatives are presented in Figure 3.

## 4. Basic Characteristics of Chitosan

### 4.1. Aqueous Solubility

CS is insoluble in water at neutral pH but it is freely soluble in slightly acidic pH due to the amine groups in its backbone. However, the solubility of CS in alkaline and neutral pH media can be enhanced by quaternization to form trimethylammonium CS derivatives. Moreover, the solubility and degradability of CS also greatly depend on the molecular weight. The CS biopolymer and its derivatives with low molecular weights and degrees of deacetylation exhibit higher aqueous solubility and degradability [55].

### 4.2. Mucoadhesion

The mucoadhesive characteristics of CS biopolymers are due to their cationic nature owing to the presence of the amine functional group. The amino and carboxyl functional groups present in the CS molecule attach to glycoproteins present on the mucus and form a hydrogen bond that leads to an adhesive effect. Mucoproteins are negatively charged molecules that attract the positively charged CS and prolong the encapsulated drug retention in the GIT, which leads to improved intestinal absorption and, thereby, oral bioavailability. The mucoadhesive characteristics of CS are strengthened under acidic and neutral pH conditions. Higher molecular weights and degrees of acetylation of CS show higher mucoadhesion [56].

### 4.3. Controlled Release

**Figure 4:**
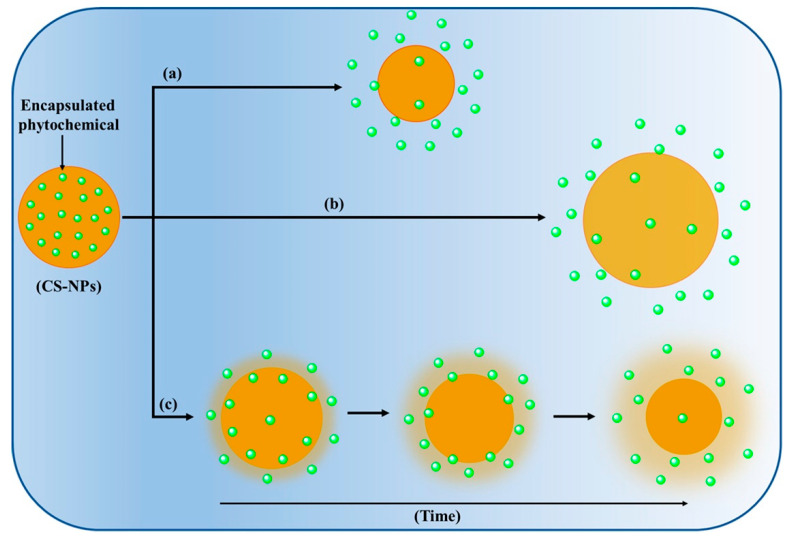
Diagrammatic representation of different controlled release mechanisms of phytochemicals from CS-NPs. (**a**) The diffusion of phytochemicals from the matrix of CS-NPs. (**b**) The swelling mechanism of phytochemical release from CS-NPs. In this mechanism, the cross-linked chain of CS absorbs a large quantity of water from the biological system without dissolving, which leads to the widening of pores, resulting in the diffusion of the encapsulated phytochemical. (**c**) The erosion mechanism of phytochemical release from CS-NPs. In the erosion process, CS loses polymer mass with time in the biological fluids, which results in controlled release of the encapsulated phytochemical from the CS-NPs.

The release of encapsulated therapeutic molecules from CS-NPs governs several release mechanisms such as swelling, diffusion, and erosion, as presented in Figure 4 [51,57]. CS-NPs exhibit initial burst release due to the fast swelling or diffusion of encapsulated molecules from the surface of the NPs. In addition, CS-NPs also exhibit a pH-dependent release of encapsulated therapeutic molecules due to the varying solubility of CS in different pH conditions. CS-NPs exhibit very low or negligible drug release in the stomach (pH 1.2) and significantly higher release in the small intestine (pH = 6.8). Furthermore, derivatization of CS further alters the release of encapsulated drug from the NPs due to the variation in the molecular weight and degree of deacetylation, and significantly influences the oral bioavailability of encapsulated therapeutic molecules [58,59]. In addition, the modification of CS-NPs by stimuli-responsive (i.e., pH) materials such as poly (propyl acrylic acid) [60] can also modulate the release of phytochemicals from CS-NPs by changing their physicochemical properties. The pH-dependent protonation and deprotonation of the amine groups of CS have been widely exploited to impart pH-responsive properties [61].

### 4.4. Intestinal Permeation Enhancement

Being a positively charged molecule, CS significantly interacts with the mucous membrane, opens the tight junctions (TJs) between epithelial cells by reducing the electrical resistance, and promotes passage via the mucosal cells, thereby improving the permeation of encapsulated drugs in CS-NPs. This mucoadhesive characteristic of CS is beneficial for the delivery of high-molecular weight compounds such as phytochemicals. On the other hand, modified CS derivatives such as thiolated and trimethyl chitosan further enhance intestinal permeation. Trimethyl chitosan presents much higher aqueous solubility and stronger mucoadhesion properties compared to CS, making it an attractive polymer as an oral drug carrier [13,55,59].

### 4.5. Biodegradability and Safety

CS is approved for diverse applications including biomedical applications by the Food and Drug Administration (FDA), being categorized as a “Generally Regarded as Safe” (GRAS) material [62]. Its non-toxic, mucoadhesive, and biodegradable characteristics are unique features supporting its application in oral drug delivery. CS and CS derivatives with a low or medium molecular weight are easily cleared from the systemic circulation by the kidney, while high-molecular weight CS is degraded into fragments before in vivo renal clearance [63].

CS and CS-based derivatives are mainly degraded by enzymes and chemical processes. CS with a high degree of deacetylation presents a higher degradation rate. Further, enzymatic catalysis directly depends on the number of amine functional groups present in the CS polymers [63]. To date, CS has generally been regarded as a non-toxic polymer and safe for oral delivery of a variety of bioactives [64]. In one study, it was revealed that CS had negligible toxicity against MCF-7 and COS7 cells compared to other compounds such as sulfide, which showed an LD_50_ of 20 µg/mL. However, it was also revealed that an increment in the charge density of CS can increase the chance of toxicity [63].

The safety and toxicity of NPs not only depend on the type of polymer used but also the size, shape, and morphology, which play an important role after oral administration. In the last two decades, phytochemical-loaded CS-NPs have been widely investigated for oral delivery, but the significant in vivo evidence on the toxicity of CS-NPs is not fully understood at present. However, a few reports on in vitro as well as in vivo models have claimed that CS-NPs are non-toxic and safe for oral administration [65].

## 5. Chitosan Nanoparticles for Oral Delivery

At present, CS-NPs are considered the most promising nanocarrier for the oral delivery of phytochemicals. As discussed elsewhere in this manuscript, CS is a non-toxic, mucoadhesive, and biodegradable polymer that is extensively investigated for improved oral bioavailability and bioactivity after encapsulation of a variety of phytochemicals in CS-based nanocarriers [66]. The potential advantages and limitations of CS-NPs over other NP-based systems for oral phytochemical delivery are summarized in Table 3. CS-NPs protect the encapsulated phytochemicals from the harsh GI milieu environment and enzymatic degradation. Further, the mucoadhesive characteristics of CS-NPs significantly prolong the residence time in the GIT, which results in improved absorption of the encapsulated molecules [67,68]. Moreover, CS can open the TJs between epithelial cells and inhibit the P-glycoprotein (P-gp) efflux transporter of epithelial cells, thus significantly facilitating the encapsulated molecules by the paracellular transport mechanism [69,70].

Recent investigations on the oral delivery of drugs revealed the strong mucoadhesive characteristics of different CS derivative-based NPs and showed their ability to improve intestinal permeation by opening TJs, which was strongly based on their degree of protonation [72,73]. The amine functional groups on CS show a pKa value of 6.5; thus, CS is protonated easily and solubilized at acidic pH, but it is aggregated at neutral pH [74,75]. This finding indicates that CS has strong mucoadhesive potential and improves the absorption of the encapsulated drug, even in a limited area (i.e., duodenum) where the pH is less or close to its pKa value [76]. Many investigations on modified CS, i.e., CS derivatives, revealed its better solubility and intestinal permeation potential at neutral pH [77,78]. The above reports suggest that CS and CS-NPs have significant potential in improving intestinal absorption and, thereby, oral bioavailability after oral administration.

## 6. Mechanism of Intestinal Absorption of Chitosan Nanoparticles

**Figure 5:**
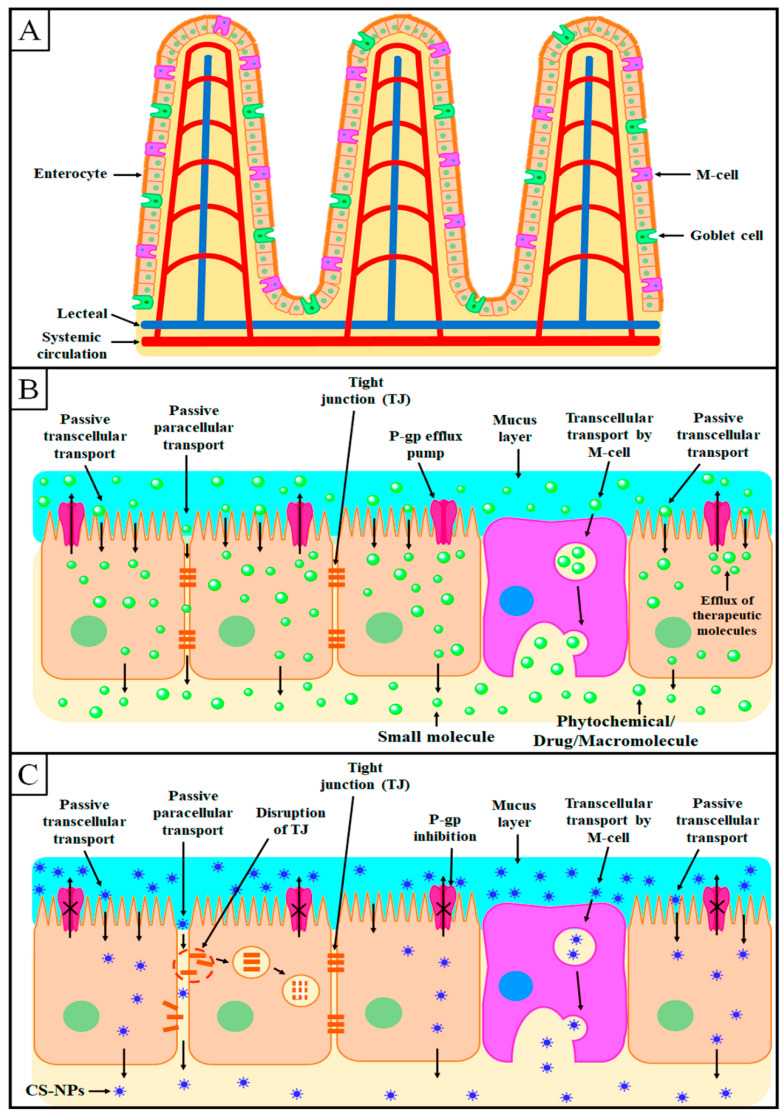
Schematic illustrations of (**A**) the structure of the intestinal epithelium, (**B**) different transport mechanisms of pure phytochemicals/synthetic drugs/macromolecules, and (**C**) different transport mechanisms of phytochemical-loaded CS-NPs.

The major barrier for oral drug administration of therapeutic molecules is the intestinal epithelium from where the drugs should be passed to reach the systemic circulation [79]. This layer consists of enterocytes, M cells, and goblet cells [80,81], as presented in Figure 5A. Enterocytes are the most abundant cells and most actively transport the therapeutic molecules/nutrients by active as well as passive transport mechanisms [82]. Goblet cells are the second most abundant cells in the intestinal epithelium that secret mucus, which is a physical barrier for pathogens [83,84]. Furthermore, M cells are characterized by flat apical surfaces that reside, in particular, in Peyer’s patches in the ileum. M cells can absorb antigens from the intestine and transport them to the systemic circulation [85].

After oral administration, the therapeutic molecules can be absorbed from the intestine by transcellular or paracellular mechanisms, as presented in Figure 5B,C [86]. As an excellent intestinal permeation enhancer, CS improves both the transcellular and paracellular transport of therapeutic molecules from the intestinal epithelium [87,88].

### 6.1. Transcellular Transport

In the transcellular transport mechanism, enterocytes and M cells play a significant role by taking the CS-NPs and mimicking the entry into the systemic circulation by transcytosis, as depicted in Figure 5C [89]. This transport mechanism can be improved by modifying the physicochemical characteristics of NPs such as particles size and mucoadhesion [90]. NPs with a size of <100 nm can be easily absorbed by enterocytes, while NPs with a size of >500 nm are absorbed by the M cells of Peyer’s patches [91,92,93].

Increasing the mucoadhesive property of NPs significantly enhances their transport across epithelial cells [94,95,96]. As discussed earlier, CS is an excellent positively charged mucoadhesive biopolymer that shows mucoadhesive properties due to its electrostatic interaction with the negatively charged sialic acid residues on the intestinal mucosa [54]. This electrostatic interaction provides a long residence time to CS-NPs on the intestinal mucosa, thereby increasing intestinal absorption [97].

### 6.2. Paracellular Transport

The paracellular transport mechanism involves the transport of therapeutic molecules from the interstitial space between the epithelial cells, as depicted in Figure 5B. Generally, the transport from the interstitial space is restricted by TJs and the narrow width between two adjacent epithelial cells. TJs allow the absorption of the required water and electrolytes but restrict the transport of other foreign agents to the systemic circulation [98,99].

As discussed in Section 5, CS can effectively open the TJs between epithelial cells, thus significantly facilitating the encapsulated molecules by the paracellular transport mechanism [69,70]. Further, the degree of deacetylation, as well as the degree of protonation, plays a major role in the intestinal permeation of CS. CS derivatives with a higher degree of deacetylation and protonation show higher intestinal permeation [72,73].

## 7. Phytochemical-Loaded CS-NPs for Improved Oral Bioavailability and Bioactivity

Here, we discuss 15 phytochemicals and their corresponding CS-NPs for improved oral bioavailability and bioactivity in different animal models. The encapsulation of phytochemicals in CS-NPs provides the advantages of improved solubility, stability in the GI milieu, controlled release profile, enhanced intestinal permeation, and, thereby, improved oral bioavailability, making their oral administration possible for the management of a variety of ailments. Consequently, small-sized CS-NPs significantly improve the bioactivity of encapsulated phytochemicals by increasing their pharmacokinetic attributes, directly targeting the target cells/tissues, and reducing extra-organ toxicity.

Apart from the above-discussed advantages of CS-NPs, the particles size (PS), polydispersity index (PDI), and zeta potential (ZP) also play a significant role in phytochemical delivery. The PS of CS-NPs is directly dependent on the CS concentration in the formulation. Moreover, CS-NPs with high-molecular weight CS show a significantly higher PS compared to CS-NPs prepared with low-molecular weight CS [71]. The absorption of NPs by epithelial cells or M cells after oral administration greatly depends on the PS of NPs, which can affect the oral absorption efficiency as well as the speed of absorption. Small-sized NPs are mainly absorbed by intestinal cells, whereas large-sized NPs are mainly absorbed by M cells [100,101]. In general, NPs with a PS of >10 nm can avoid renal clearance and permeate into tissues, although NPs with a PS of 10–20 nm can be extensively distributed in different organs via tight endothelial connections. NPs with a PS of >200 nm can be quickly engulfed by the mononuclear phagocytic system and accumulate in the liver and spleen [102]. Therefore, the PS of CS-NPs should be optimum for the effective oral delivery of phytochemicals. The PDI represents the homogeneity of CS-NPs. The lower the PDI, the better the uniformity of NPs, which also suggests a uniform distribution of NPs. A low PDI of CS-NPs represents good colloidal stability [103] and is one of the necessary requirements for the development of efficient phytochemical-loaded CS-NPs. In one study, Du et al. developed TMCS-coated nanostructured lipid carriers for improved oral delivery of kaempferol. The developed CS-based NPs showed a PS of less than 125 nm, with an excellent PDI value of <0.3. A PDI value of less than 0.3 represents excellent homogeneity among NPs and excellent stability in different temperature conditions. Furthermore, the developed CS-based NPs exhibited significantly higher intestinal permeation and almost two times higher oral bioavailability in SD rats compared to uncoated NPs [104]. On the other hand, the ZP is also an important parameter of CS-NPs that greatly affects their colloidal stability as well as their mucoadhesive characteristics [105,106]. A high ZP (>±20) of CS-NPs represents high stability [107,108]. The ZP significantly increases with an increase in the CS concentration during the development of CS-NPs. In addition, CS derivative-based NPs such as TMCS-based NPs showed a significantly higher ZP, meaning they had excellent stability and mucoadhesive characteristics [109,110]. In another study, Abd El Hady et al. developed CS-modified polymeric NPs for improved oral delivery of diosmin. In their study, it was found that the increase in the concentration of CS significantly increased the PS from 334 to 862 nm, and the ZP from +27 to +33 mV, in the developed NPs. Moreover, the developed CS-based NPs exhibited excellent colloidal stability and mucoadhesive properties [111]. To date, extensive investigations have been conducted to evaluate the oral bioavailability and bioactivity of phytochemical-loaded CS-NPs in the management of different diseases [112,113]. The major pharmacokinetic attributes and bioactivity obtained after the encapsulation of the selected phytochemicals in CS-NPs are explained below and summarized in Table 4.

### 7.1. Curcumin

Curcumin (CUR), a naturally abundant orally active phytochemical, is mostly obtained from the rhizomes of Curcuma longa. CUR has a long history of utilization as a food additive and natural remedy in the management of different diseases, particularly in India and China. CUR shows strong antioxidant, anti-inflammatory, and anticancer activity [114,115,116]. Clinical trials on CUR have demonstrated its non-toxicity, with negligible adverse drug reactions [117]. Unfortunately, the clinical application of CUR is very limited due to its limited aqueous solubility, instability in light and the GI milieu, poor intestinal absorption, and low bioavailability after oral administration [118]. Therefore, many researchers across the world have tried hard to improve its aqueous solubility and oral bioavailability by encapsulating it in CS-NPs. In this context, Raja et al. developed hydrophobic acrylonitrile (AN)-modified chitosan (CS)–arginine (AR)-conjugated CUR-NPs (AN-CS-AR/CUR-NPs) and evaluated their pharmacokinetic attributes in Sprague Dawley rats [119]. The developed NPs exhibited excellent mucoadhesive properties and sustained release of CUR for up to 72 h in simulated gastric fluids (SGF, pH 2.0) as well as in simulated intestinal fluids (SIF, pH 6.8). Moreover, cell culture studies revealed the much higher cellular internalization and cytotoxicity against HT-29 cells of AN-CS-AR/CUR-NPs compared to free CUR in a dose-dependent manner. Furthermore, pharmacokinetic evaluation in Wistar rats revealed that the AN-CS-AR/CUR-NPs showed more than 4.5 times enhanced oral bioavailability compared to free CUR. In another study, Ng and associates developed CUR-loaded CS-NPs (CUR-CS-NPs) for improved oral efficacy against feline infectious peritonitis virus [120]. CUR-CS-NPs revealed negligible toxicity against Crandell–Rees feline kidney cells and at least three times higher antiviral efficacy compared to free CUR. Furthermore, CUR-CS-NPs exhibited more than 2.6 times higher oral bioavailability after single-dose oral administration in young cats compared to free CUR.

Khatik and associates developed eudragit (ET)-modified CS-NPs for colon-targeted delivery of CUR to improve oral bioavailability [121]. ET-CUR-CS-NPs exhibited sustained release of CUR in both gastric and intestinal fluids up to 24 h of study. Cell culture studies on Caco-2 cell lines revealed that the ET-CUR-CS-NPs exhibited significantly higher cellular internalization and cytotoxicity compared to free CUR. Moreover, the histopathological study revealed the excellent colon-targeting potential of ET-CUR-CS-NPs due to the mucoadhesive characteristics of CS. Furthermore, ET-CUR-CS-NPs exhibited more than 4-fold improved oral bioavailability compared to free CUR in Wistar rats. In another study, Ramalingam and Ko developed CS-modified solid lipid nanoparticles (CS-SLNs) for improved oral delivery of CUR [122]. The developed formulation was mainly evaluated for its oral bioavailability and brain distribution. CS-CUR-SLNs exhibited sustained release and significantly higher in vitro dissolution of CUR. Moreover, CS-CUR-SLNs exhibited significantly higher cytotoxicity against MCF-7 and B16F10 cells. Furthermore, CS-CUR-SLNs revealed 23.07 times higher oral bioavailability and a much higher concentration of CUR in the brain compared to free CUR after oral administration in Balb/c mice. Similarly, Beak and Cho developed N-carboxymethyl chitosan (NCCS)-coated SLNs (NCCS-SLNs) for improved oral bioavailability of CUR [123]. Initial burst release followed by sustained release of CUR for up to 24 h was observed with NCCS-CUR-SLNs. Moreover, cell culture studies revealed the improved cellular uptake and multiple-fold higher cytotoxicity against MCF-7 cells of NCCS-CUR-SLNs compared to free CUR. Furthermore, the NCCS-CUR-SLNs exhibited 6.3 and 9.5 times higher lymphatic uptake and oral bioavailability compared to free CUR in Sprague Dawley rats. Based on the above in vitro and in vivo investigations, CUR-loaded CS-NPs might be an excellent delivery system to improve oral bioavailability and bioactivity.

### 7.2. Quercetin

Quercetin (QRT) is an orally active but highly lipophilic bioactive phytochemical, widely distributed in many fruits and vegetables. QRT has various strong health-promoting activities such as anticancer and anti-inflammatory activities and reduces the complications of heart-related diseases [124,125]. Its therapeutic efficacy in different diseases is mainly attributed to its excellent antioxidant activity [126,127]. Nevertheless, its clinical application in the pharmaceutical field is still limited due to its low oral bioavailability [127,128]. QRT’s poor aqueous solubility and degradation during the digestion process after oral administration are the major factors behind its poor oral bioavailability [127,128,129]. In addition, the therapeutic application of QRT is also restricted due to its instability at room temperature, oxidation, and degradation at acidic pH (i.e., stomach, pH 1.2) [130]. To overcome these challenges, Barbosa et al. fabricated pH-responsive fucoidan (FD)/CS-NPs for improved oral absorption of QRT in the GIT [131]. FD/CS-QRT-NPs showed almost 80% drug release in gastric fluids after 3 h of the study, which confirmed the pH-responsive drug release profile. Furthermore, the developed NPs revealed a strong and dose-dependent antioxidant potential compared to the free drug. In another study, Zhou et al. developed QRT-loaded caseinate–CS double-layered zein NPs for improved oral delivery [132]. Freeze-dried NPs revealed excellent colloidal as well as storage stability. The developed NPs revealed excellent mucoadhesive properties and sustained release of QRT in SGF for up to 3 h. After single-dose oral administration of NPs in Sprague Dawley rats, 1.89-fold higher oral bioavailability than free QRT was observed. Similarly, Ma et al. developed a zein/CS-stabilized nanoemulsion for improved oral bioavailability of QRT [133]. The developed NPs showed excellent (77.41%) in vitro digestion and bioaccessibility (60%) compared to free QRT. Cell culture studies in Caco-2 cells revealed the significantly higher cellular internalization and cytotoxicity of the NPs compared to free QRT. Furthermore, the NPs revealed 41.22% higher oral bioavailability than free QRT. In another study, Mukhopadhyay et al. developed QRT-loaded succinylated CS/alginate NPs for improved oral efficacy against diabetes [134]. The pharmacodynamic study in diabetic Wistar rats suggested the better hypoglycemic activity of the NPs compared to the free drug after oral administration. Furthermore, the histological study suggested that the NPs were non-toxic and safe for oral treatment of diabetes. Similarly, Tzankova et al. developed CS/alginate NPs and evaluated their hepatoprotective and antioxidant activity for the management of paracetamol-induced toxicity after oral administration [135]. The developed NPs were found to be safe after cytotoxicity evaluation in human hepatoma HepG2 cells. The developed NPs significantly decreased the levels of serum transaminases ALT and AST, attenuated lipid peroxidation, and restored the levels of glutathione compared to the free drug after oral treatment for 7 days at the dose of (0.18 mg/kg). Therefore, it was suggested that the developed NPs have significant potential in terms of hepatoprotective efficacy and antioxidant activity. From the above-discussed pre-clinical investigations of QRT, it can be concluded that CS-NPs can be considered an alternative nanoplatform for the effective delivery of QRT.

### 7.3. Resveratrol

Resveratrol (RVT) is a natural non-flavonoid polyphenol mainly obtained from *Polygonum cuspidatum*, grapes, and peanuts. An extensive body of literature suggests that it has various strong therapeutic activities against cancer, inflammation, CNS-related disorders, and cardiovascular and hepatic diseases [136,137,138]. Despite its excellent biological activities, the clinical application of RVT is restricted because of its poor aqueous solubility, high lipophilicity, low oral bioavailability, and rapid metabolism and clearance from the systemic circulation [139,140]. Therefore, improvement in RVT’s aqueous solubility and intestinal absorption is a prerequisite to improve its oral bioavailability and, thereby, bioactivity. In this context, Zu et al. developed RVT-loaded carboxymethyl CS-NPs (RVT-CMCS-NPs) for improved oral bioavailability [141]. RVT-CMCS-NPs revealed better in vitro dissolution in SGF (pH 1.2) with 81.3% drug release in 68 h, in a sustained manner. Furthermore, RVT-CMCS-NPs revealed almost 3.5 times higher oral bioavailability in SD rats than free RVT. In another study, Ramalingam and Ko developed RVT-loaded N-trimethyl chitosan–g-palmitic acid surface-modified SLNs (RVT-NTMCS-SLNs) and evaluated their biocompatibility, toxicity, and oral bioavailability [142]. A biocompatibility study on NIH/3T3 cell lines suggested that the RVT-NTMCS-SLNs were biocompatible and non-toxic. Furthermore, the in vivo pharmacokinetic study in Balb/c mice after single-dose (25 mg/kg) oral administration revealed that the RVT-NTMCS-SLNs exhibited 3.8 times higher bioavailability than free RVT.

Du et al. investigated the oral efficacy of RVT-loaded CS-NPs (RVT-CS-NPs) against streptozotocin-induced diabetes mellitus in Wistar rats [143]. RVT-CS-NPs exhibited dose-dependent in vitro α-glucosidase and α-amylase inhibition. The maximum α-glucosidase (77.32%) and α-amylase (78.4%) inhibition was observed at 500 µg/mL. Furthermore, oral administration of RVT-CS-NPs revealed a marked reduction in blood glucose levels in Wistar rats with diabetes mellitus and maintained the lipid profiles. In another study, Pauluk et al. developed RVT-loaded CS-coated zein NPs (RVT-CS-ZNPs) for improved oral delivery [144]. RVT-CS-ZNPs exhibited excellent colloidal stability in SGF as well as in SIF. RVT-CS-ZNPs exhibited significantly higher mucoadhesive properties compared to free RVT. Furthermore, after assessing the antioxidant activity by the ABTS assay, it was revealed that the RVT-CS-ZNPs exhibited a comparable antioxidant potential in respect to the free drug. Therefore, the findings from the above pre-clinical reports indicate that RVT-loaded CS-NPs can be a potential oral nanocarrier to achieve better therapeutic efficacy.

### 7.4. Thymoquinone

Thymoquinone (THQ) is a naturally occurring bioactive phytochemical mainly extracted from Nigella sativa. THQ is a highly lipophilic yellow crystalline phytochemical that has very limited aqueous solubility but exceptional efficacy against almost all diseases [145]. Despite its excellent therapeutic efficacies, the clinical application of THQ is very limited due to its poor aqueous solubility, high lipophilicity, instability in the GI milieu, and low oral bioavailability. Hence, a high oral dose, as well as frequent dosing, is required to achieve optimum therapeutic efficacy [146,147]. In one study, Rahat et al. developed THQ-loaded CS-coated SLNs (THQ-CS-SLNs) and evaluated their pharmacokinetic attributes in Wistar rats after oral administration [148]. The THQ-CS-SLNs revealed 4.05 times higher intestinal permeation compared to the free drug suspension. The improved intestinal permeation was further confirmed by confocal microscopy. Furthermore, the THQ-CS-SLNs showed 3.53 times higher oral bioavailability after single-dose oral administration compared to free THQ. Similarly, the same researchers developed THQ-loaded CS-modified polycaprolactone NPs (THQ-CS-PCL-NPs) and evaluated them for intestinal permeation as well as oral bioavailability in Wistar rats [149]. THQ-CS-PCL-NPs showed excellent mucoadhesive properties and 5.34 times higher intestinal permeation compared to the free drug suspension. Furthermore, the THQ-CS-PCL-NPs exhibited 4.16 times higher relative oral bioavailability after single-dose oral administration compared to the free THQ. In another study, Fakhria et al. developed THQ-loaded chitosan vesicles (THQ-CS-V) for improved oral efficacy against hyperglycemia [150]. The developed THQ-CS-V exhibited about 1.9-fold higher intestinal permeation and much higher mucoadhesive efficiency compared to the free THQ suspension. Furthermore, in vivo thermodynamic investigation in Wistar rats revealed much better activity in terms of normalizing lipid profiles and liver function parameters compared to free THQ. The overall results suggest that THQ-loaded CS-NPs might be a useful delivery system for improved oral delivery.

### 7.5. Epigallocatechin-3-Gallate

Epigallocatechin gallate (EGCG) is a naturally occurring flavonoid particularly present in green tea [151,152]. EGCG shows excellent therapeutic efficacy against various diseases including cancer, viral diseases, and inflammation, as well as neuro- and cardioprotection [153,154,155]. However, it is unstable in neutral as well as basic conditions, and it undergoes rapid degradation by deprotonation of the alcoholic functional group present in its skeleton. Hence, it exhibits poor bioactivity after oral administration [156,157]. EGCG showed very poor intestinal absorption, and only 5% of it reached the systemic circulation after oral administration in rats [158,159]. In addition, EGCG is metabolized very fast in the systemic circulation and converted into different by-products that are eliminated rapidly [160,161]. Hence, an efficient nanocarrier for EGCG delivery should be developed to achieve good therapeutic efficacy. In this regard, Khan et al. developed EGCG-loaded CS-NPs (EGCG-CS-NPs) for improved oral efficacy against prostate cancer [162]. The in vivo antitumor activity in 22Rν1 tumor-bearing athymic nude mice after oral treatment with EGCG-CS-NPs exhibited significantly higher tumor inhibition and prostate-specific antigen levels compared to free EGCG. Similarly, Siddiqui et al. prepared EGCG-CS-NPs for improved oral efficacy in the management of melanoma [163]. The developed EGCG-CS-NPs exhibited more than 8-fold higher cytotoxicity against Mel 928 cells compared to the free drug. Furthermore, after oral administration, EGCG-CS-NPs exhibited significantly higher antitumor efficacy in Mel 928 tumor-bearing athymic nude mice. In another study, Hong et al. developed EGCG-loaded CS–polyaspartic acid (PPA) NPs (EGCG-CS/PAA-NPs) for improved oral efficacy against atherosclerosis [164]. EGCG-CS/PAA-NPs revealed excellent colloidal stability and sustained release of EGCG in gastrointestinal fluids. Furthermore, in an in vivo efficacy study in New Zealand white rabbits, EGCG-CS/PAA-NPs revealed much higher efficacy compared to the free drug. Further, Dube et al. developed EGCG-CS-NPs to enhance the plasma drug concentration after oral administration in Swiss outbred mice [165]. The results revealed that the developed EGCG-CS-NPs exhibited a 1.5 times higher plasma concentration of the drug compared to the free drug. Furthermore, EGCG-CS-NPs exhibited a 2.3-fold improved plasma concentration of the drug after exposure to the jejunum compared to the free EGCG. Therefore, it can be concluded that CS-NPs might be an ideal biodegradable nanocarrier for improved oral bioavailability and bioactivity of EGCG.

### 7.6. Ursolic Acid

Ursolic acid (UA) is a naturally occurring pentacyclic triterpenoid that belongs to the cyclosqualenoid family. UA is abundantly found in various foods and herbs [166]. Extensive pre-clinical and clinical investigations on UA have suggested a wide range of therapeutic applications such as antidiabetic, anticancer, antiulcer, and antihepatitis drugs, and demonstrated its negligible toxicity and side effects [167,168,169]. However, the prescribed formulations of UA are not available in the market because of their physicochemical characteristics. UA is a highly lipophilic compound and shows very low aqueous solubility that is responsible for its low bioavailability and, thereby, bioactivity after oral administration [170]. To address these challenges, Antonio et al. developed UA-loaded CS-coated poly (lactic acid) NPs (UA-CS-PLA-NPs) to improve oral bioavailability [171]. The UA-CS-PLA-NPs showed excellent hemocompatibility, and the CS coating of NPs resulted in multiple-fold improved mucoadhesiveness to mucin particles compared to the uncoated NPs. Furthermore, in an in vivo pharmacokinetic study in Wistar rats, UA-CS-PLA-NPs revealed 3.83- and 4.14-fold higher relative oral bioavailability and biological half-life compared to the free drug after single-dose administration. In another study, Das et al. developed UA-loaded CS-coated nanostructured lipid carriers (UA-CS-NLCs) for improved oral efficacy against visceral leishmaniasis [172]. The UA-CS-NLCs showed much higher ex vivo drug uptake by macrophages compared to the free drug. Furthermore, the in vivo antileishmanial efficacy experiment on Balb/c mice revealed more than 98% parasite suppression for UA-CS-NLCs compared to the free drug after oral administration. Wang et al. developed UA-loaded CS-modified liposomes (UA-CS-L) for improved oral efficacy against cervical tumors [173]. The in vitro cell culture study using Hela cells revealed that UA-CS-L exhibited a 76.46% Hela cell inhibition rate compared to free UA. Moreover, the in vivo antitumor study and biodistribution in U14 cervical cancer-bearing CD-1 female mice revealed that UA-CS-L exhibited a 61.26% tumor inhibition rate and much higher UA accumulation in tumors compared to the free drug. Furthermore, histological examination of tumors treated orally with UA-CS-L revealed the better potential for killing cancer cells. From the above-discussed findings, it can be inferred that CS-NPs significantly improve the intestinal permeation of encapsulated UA. The enhancement of UA in CS-NPs significantly improves intestinal permeation, which leads to higher bioavailability and therapeutic activity.

### 7.7. Ferulic Acid

Ferulic acid (FA) is a naturally occurring phenolic phytocompound that is abundantly found in cereals, citrus fruits, and beverages. Extensive pre-clinical and clinical investigations on UA have suggested its wide range of therapeutic potentials such as antioxidant, antidiabetic, anticancer, and pulmonary protective activity [174,175,176,177]. Moreover, its low toxicity makes it a potential candidate in food as well as in the pharmaceutical field [178]. Nevertheless, its clinical application in the pharmaceutical field is still limited due to its low oral bioavailability, fast metabolism, and elimination after oral administration [179]. In one study, Telange et al. developed FA–phospholipid complex-loaded CS-NPs (FA-PLC-CS-NPs) for improved oral bioavailability in Wistar rats [180]. The developed FA-PLC-CS-NPs exhibited almost 2-fold higher intestinal permeation compared to the free drug solution. Moreover, the FA-PLC-CS-NPs showed much higher antioxidant activity in CCl_4_-intoxicated Wistar rats, by restoring the elevated marker enzymes in the FA-CS-NPs. Furthermore, FA-PLC-CS-NPs revealed almost 2.4-fold higher relative oral bioavailability after single-dose (20 mg/kg) oral administration in Wistar rats compared to the FA-CS-NPs. In another study, Lima et al. developed FA-loaded CS-coated PLGA-NPs (FA-CS-PLGA-NPs) for enhanced intestinal absorption of FA [181]. The in vitro antioxidant activity and cell culture study with B16-F10 and HeLa cells revealed that the FA-CS-PLGA-NPs showed comparable effects compared to the free FA. On the other hand, the intestinal permeability study using Caco-2 monolayers revealed more than 3-fold higher permeation with FA-CS-PLGA-NPs compared to the free FA. From the above discussion on pre-clinical reports, biodegradable CS-NPs have been reported to enhance the bioavailability and biological activity of FA.

### 7.8. 10-Hydroxycamptothecin

10-Hydroxycamptothecin (HCPT; SN38) is a potent derivative of camptothecin, which is a natural quinoline alkaloid obtained from the bark and stems of *Camptotheca acuminate* [182]. HCPT is one of the most effective drugs to treat colorectal cancer. However, oral treatment with conventional SN38 formulations is very limited due to its low solubility, which results in poor intestinal permeation and, thereby, low bioavailability (8%) after oral administration. In addition, SN38 shows dose-related gastrointestinal toxicity [183,184]. In one study, Sharifi et al. developed SN38-loaded CS/hyaluronic acid (HA)-modified NPs (SN38-CS/HA-NPs) and evaluated their oral bioactivity against colon cancer [185]. The in vitro cell culture study using Caco-2 cancer cells revealed that the SN38-CS/HA-NPs exhibited significantly higher cellular internalization as well as cytotoxicity compared to the free SN38. Furthermore, the in vivo antitumor efficacy study in Balb/c mice bearing C26 tumors further confirmed the higher therapeutic efficacy of SN38-CS/HA-NPs compared to the free SN38. In another study, Guo et al. investigated the mechanism of intestinal absorption of SN38-loaded CS-coated PLGA-NPs after oral administration in Sprague Dawley rats [186]. SN38-CS-PLGA-NPs revealed significantly higher intestinal absorption compared to the free drug due to the inhibition of the P-gp efflux transporter by CS and PLGA. Furthermore, the cell culture study using Caco-2 cells revealed that the SN38-CS-PLGA-NPs exhibited significantly higher cellular uptake and comparable cytotoxicity compared to the free SN38. The researchers concluded that the SN38-CS-PLGA-NPs were absorbed via clathrin-mediated endocytosis and entered into the systemic circulation. Therefore, the above findings suggest the excellent ability of CS-NPs for SN38 delivery in the improvement in bioavailability and bioactivity after oral administration.

### 7.9. Apocynin

Apocynin (APN) is a natural organic compound extracted from the roots of either *Apocynum cannabinum* or *Picrorhiza kurroa*, which are native to the Western Himalayas [187]. APN is used in the treatment of a variety of diseases. Various reports have suggested the good therapeutic potential of APN in different ailments such as atherosclerosis, asthma, cancer, cardiovascular and neurodegenerative diseases, and inflammatory bowel disease [188]. Nevertheless, its clinical application is limited due to its low water solubility and oral bioavailability (<10%), rapid renal clearance, and high protein binding [189]. In one study, Aman et al. developed and optimized APN-loaded CS-modified SLNs (APN-CS-SLNs) for improved bioresidence and oral bioavailability [190]. The in vivo pharmacokinetic study of APN-CS-SLNs revealed 2.52 times improved relative oral bioavailability after single-dose (14 mg/kg) oral administration compared to the free drug in Sprague Dawley rats. In another study, the same research group developed APN-loaded CS oligosaccharide-based mucoadhesive NPs (APN-CSOS-NPs) for improved oral efficacy against gastric ulcers [191]. APN-CSOS-NPs revealed much higher antiulcer efficacy on ketoprofen-induced gastric ulcers of Wistar albino rats than the free APN after oral administration. The superior in vivo therapeutic efficacy of APN-CSOS-NPs was further confirmed by histological as well as biochemical studies. Overall, it can be concluded that CS-NPs open a new vista in the improvement in the bioavailability and bioactivity of encapsulated APN after oral administration.

### 7.10. Astaxanthin

Astaxanthin (ATX) is a carotenoid mainly obtained from different marine plants and animals. ATX shows excellent therapeutic potential with antioxidant, anti-inflammatory, antidiabetic, antiobesity, and anticancer activity. Furthermore, it shows strong antioxidant activity by inhibiting biomarkers for oxidation [192]. Some studies have suggested that ATX can exhibit 10 to 500 times higher antioxidant potential compared to β-carotene and vitamin E [193]. However, its extremely low aqueous solubility and poor oral bioavailability limit its clinical application. In addition, its instability in acidic pH conditions and degradation during storage and digestion cause a significant reduction in its antioxidant activity [194]. In one study, Zhu et al. developed ATX-loaded poly (ethylene glycol) (PEG) engineered CS-NPs (ATX-PEG-CS-NPs) to improve its intestinal permeation and, thereby, oral bioavailability [195]. The ex vivo single-pass perfusion study revealed that the ATX-PEG-CS-NPs exhibited excellent intestinal permeability, especially in the jejunum, compared to free ATX. Furthermore, in the in vivo pharmacokinetic study in Sprague Dawley rats after single-dose (8 mg/kg) administration, ATX-PEG-CS-NPs revealed more than six times improved relative oral bioavailability compared to the free drug. In another study, Hu et al. developed and optimized ATX-loaded CS–caseinate–dextran ternary complex NPs for improved oral bioactivity [196]. The developed NPs exhibited significantly higher dose-dependent antioxidant activity compared to the free drug. Further, the NPs revealed excellent biocompatibility and did not show any significant toxicity to the LX-2 cells in the cell culture study. Furthermore, ATX-loaded NPs exhibited significantly enhanced antifibrogenic efficacy against LX-2 cells compared to free ATX. This activity was confirmed by lowering the expression level of the TGFβ1-induced fibrogenic gene and COL1A1 protein levels. Therefore, the above pre-clinical investigations suggest that CS-NPs hold a promising ability to improve the bioavailability and biological activity of encapsulated ATX after oral administration.

### 7.11. Berberine

Berberine (BER) is a naturally occurring alkaloidal phytochemical that is mainly used in the management of diarrhea due to its antimotility and antisecretory action. In addition, BER shows good therapeutic activity against a variety of diseases such as cancer, HIV, diabetes, inflammation, convulsion, aging, and pain [197,198]. Unfortunately, the clinical application of BER is restricted due to its limited aqueous solubility, poor intestinal absorption, and low bioavailability after oral administration. In addition, BER shows side effects such as anaphylactic rash and drug rash after intramuscular as well as intravenous administration [199,200]. In one study, Nguyen et al. developed BER-loaded CS-coated liposomes (BER-CS-L) for improved oral delivery [201]. BER-CS-L displayed excellent stability and slower drug release compared to the uncoated BER-L. Furthermore, the in vivo oral pharmacokinetic study in New Zealand rabbits revealed that the BER-CS-L exhibited 2.86 and 1.55 times improved relative oral bioavailability compared to free BER and uncoated BER-L. In another study, Wu et al. developed BER-loaded CS and fucoidan-based NPs for defective intestinal epithelial tight junction barrier treatment [202]. A higher intestinal permeation with the NPs was observed compared to the free drug in Caco-2 cells/RAW264.7 cells. In addition, the NPs significantly protected the intestinal tight junction barrier function by reducing nitric oxide and inflammatory cytokine levels. Therefore, CS-NPs hold promising potential to increase the overall oral bioavailability of BER by improving its mucoadhesion and intestinal permeation through the opening of TJs.

### 7.12. Piperine

Piperine (PPN) is a natural nitrogen-containing alkaloidal phytochemical obtained from the fruits of *Piper nigrum*, *Piper longum*, and other piper species of the Piperaceae family [203,204]. PPN shows significant therapeutic efficacy against almost all diseases. Nevertheless, its clinical application is limited due to its low water solubility, instability in acidic pH conditions, pre-systemic metabolism, poor intestinal absorption, and oral bioavailability (24%) [205,206]. In one study, Zafar et al. developed PPN-loaded CS-coated nanostructured lipid carriers (PPN-CS-NLCs) to enhance oral bioavailability as well as bioactivity [207]. The developed PPN-CS-NLCs exhibited excellent mucoadhesive properties, stability in the gastrointestinal media, and controlled release of PPN for up to 24 h. Further, PPN-CS-NLCs showed more than 10 times higher ex vivo intestinal permeation than free PPN. Moreover, the in vivo oral pharmacokinetic study in Wistar rats revealed that the PPN-CS-NLCs exhibited 3.76 times higher oral bioavailability than free PPN. Furthermore, PPN-CS-NLCs showed strong antidiabetic efficacy in streptozotocin-induced diabetic rats compared to the free PPN. In conclusion, PPN-loaded CS-NPs can be an ideal nanoplatform to achieve multiple-fold higher bioavailability and bioactivity after oral administration.

### 7.13. Lutein

Lutein (LTN) is a dihydroxy carotenoid with strong antioxidant activity [208]. LTN has significant therapeutic potential against different cancers, atherosclerosis, and age-related macular degeneration [209]. However, LTN is a highly lipophilic phytochemical that undergoes extensive oxidation when exposed to oxygen, light, and heat [210,211]. In addition, LTN is almost insoluble in water, is unstable in the GI milieu, has poor intestinal permeation, and shows poor oral bioavailability (3–13%) [212]. These factors limit the clinical application of LTN. In one study, Toragall et al. developed CS–oleic acid–sodium alginate-based hybrid NPs for LTN (LTN-CHNPs) to improve its oral bioavailability [213]. The LTN-CHNPs exhibited more than a 1000-fold higher solubility improvement in LTN and showed a strong mucoadhesive property. Moreover, LTN-CHNPs showed negligible cytotoxicity on Caco-2 cells and significantly higher in vitro intestinal transport in Caco-2 monolayers, suggesting the compatibility of the NPs. Furthermore, the in vivo oral pharmacokinetic study using Wistar rats revealed that the LTN-CHNPs exhibited 128.3% improved oral bioavailability compared to free LTN. In another study, Shwetha et al. developed LTN-loaded CS and phosphatidylcholine-based NPs (LTN-CS-PPT-NPs) for improved oral delivery [214]. The LTN-CS-PPT-NPs revealed excellent drug release profiles in both SGF and SIF for up to 42 h of study. Furthermore, the NPs exhibited multiple-fold higher intestinal permeation across Caco-2 monolayers compared to the free drug. As per the pre-clinical findings in the above-discussed reports, it can be concluded that CS-NPs can be a potential nanocarrier to improve the oral bioavailability and bioactivity of LTN.

### 7.14. Silymarin

Silymarin (SIL) is a flavonoid-like phytocompound extracted from the fruits of *Silybum marianum L* [215]. SIL shows strong therapeutic potential with its hepatoprotective, antioxidant, anti-inflammatory, anticancer, and hypolipidemic effects [215,216]. However, SIL has extremely low aqueous solubility that limits its intestinal absorption (23–47%), and thereby extremely low oral bioavailability (0.73%) [217,218]. These factors limit the clinical application of SIL. In one study, Liang et al. developed SIL-loaded CS-modified lipid–polymer hybrid NPs (SIL-CS-LPHNPs) for improved oral bioavailability and lipid-lowering efficacy [219]. SIL-CS-LPHNPs exhibited significantly improved intracellular uptake and lipid disposition in HepG2 cells compared to the free drug. Moreover, SIL-CS-LPHNPs revealed 14.38-fold improved relative bioavailability after single-dose (20 mg/kg) oral administration in Wistar rats compared to the drug suspension. Furthermore, SIL-CS-LPHNPs showed significantly reduced blood lipid levels (TG), improved liver function (AST and ALT), and reduced lipid accumulation in the livers after oral administration into I148M transgenic mice compared to the free drug suspension. In another study, Aboshanab et al. developed SIL-loaded CS-NPs (SIL-CS-NPs) for improved oral efficacy against liver disease [220]. In vivo biochemical studies in Sprague Dawley rats revealed that the SIL-CS-NPs exhibited a significant increment in serum AST, ALT, ALP, and GGT compared to free SIL after oral administration. Further, SIL-CS-NPs improved blood lipid profiles compared to the free drug after oral administration. Furthermore, histological examination further confirmed the therapeutic efficacy and safety of the SIL-CS-NPs after oral administration. Therefore, the above pre-clinical investigations suggest CS-NPs hold a promising ability to enhance the oral bioavailability of SIL in comparison to the free drug.

### 7.15. Naringenin

Naringenin (NGN) is a natural flavonoid that has a wide range of therapeutic potentials such as antioxidant, hepatoprotective, anti-inflammatory, antiatherogenic, antifibrogenic, and anticancer effects, with negligible toxicity to normal healthy cells [221,222,223]. Unfortunately, the clinical application of NGN is restricted due to its limited aqueous solubility (46 ± 6 μg/mL), instability in the GI milieu, pre-systemic metabolism, poor intestinal absorption, and low bioavailability (5.81%) after oral administration [224,225]. In one study, Maity et al. developed NGN-loaded alginate (ALG)-coated CS-NPs (NGN-ALG-CS-NPs) for improved oral efficacy against diabetes mellitus [226]. NGN-ALG-CS-NPs showed excellent mucoadhesive properties and sustained release of NGN for up to 24 h. The in vivo anti-hypoglycemic efficacy study in streptozotocin-induced diabetic Wistar rats revealed the significantly higher efficacy after oral administration of NGN-ALG-CS-NPs compared to the free drug. Furthermore, the in vivo histopathological examination and blood parameters revealed the excellent safety of the developed NPs. In another study, Kumar et al. developed NGN-CS-NPs for improved oral delivery and cytotoxicity in lung cancer cells [227]. The in vitro release experiment revealed almost 15% NGN release in SGF (pH 1.2) after 180 min of study. Moreover, NGN-CS-NPs showed a significantly strong antioxidant potential compared to the free drug. Furthermore, the cell culture study revealed the non-toxic properties of NGN-CS-NPs on normal fibroblast 3T3 cells and their significantly higher cytotoxicity against A549 lung cancer cells. The in vitro and in vivo investigations revealed that CS-NPs might be an excellent delivery system for NGN to improve its oral bioavailability and therapeutic efficacy.

**Table 4:** Summary of major outcomes after oral administration of selected phytochemicals encapsulated in CS-NPs.

Phytochemical	Main Excipients	PS (nm)	ZP (mV)	EE (%)	Major Outcome	Ref
Curcumin	Chitosan, acrylonitrile, arginine	218.3 ± 7.2	40.1 ± 2.81	76.53 ± 3.58	Excellent mucoadhesion Higher cellular uptake and cytotoxicity against HT-29 cells than free drug 4.5-fold higher bioavailability after oral administration in Sprague Dawley rats	[119]
	Chitosan	332.4 ± 9.4	42.1 ± 3.0	77.2 ± 3.6	3-fold higher antiviral activity than free drug 2.6-fold higher bioavailability after oral administration in cats	[120]
	Chitosan, eudragit	236 ± 3.2	–29.8 ± 2.2	42 ± 1.9	Significantly higher cellular internalization in Caco-2 cell monolayers than free drug Much higher colon-targeting potential due to improved mucoadhesion More than 4-fold improved bioavailability after oral administration in Wistar rats	[121]
	N-trimethyl chitosan, palmitic acid, TPGS	311.9 ± 67.7	35.7 ± 1.03	93.12 ± 0.08	Significantly higher cytotoxicity against MCF-7 and B16F10 cells than free drug 23.07-fold improved bioavailability after oral administration in Balb/c mice	[122]
Quercetin	Chitosan, caseinate, zein	~550	55	>78	Excellent mucoadhesive properties 1.89-fold improved bioavailability after oral administration in Sprague Dawley rats	[132]
	Chitosan, zein	~100 nm	~60	>90	Excellent in vitro digestion and bioaccessibility Significantly higher cellular uptake and cytotoxicity against Caco-2 cells than free drug 41.22% improved oral bioavailability	[133]
Resveratrol	Carboxymethyl chitosan	155.3 ± 15.2	–10.2 ± 6.4	44.5 ± 2.2	Controlled release of encapsulated phytochemical ~3.5-fold improved bioavailability after oral administration in Sprague Dawley rats	[141]
	N-trimethyl chitosan, palmitic acid	258.2 ± 18.7	20.7 ± 0.63	95.45 ± 2.18	Excellent biocompatibility in NIH/3T3 cell lines 3.8-fold improved bioavailability after oral administration in Balb/c mice	[142]
Thymoquinone	Chitosan, glyceryl monostearate	166.5 ± 5.83	12.5 ± 1.21	82.66 ± 3.47	Significantly higher mucoadhesion and gastrointestinal retention 4.05-fold improved intestinal permeation 3.53-fold improved bioavailability after oral administration in Wistar rats	[148]
	Chitosan, phospholipid	372.2 ± 3.11	13.12 ± 2.3	81.38 ± 3.85	1.9-fold higher intestinal permeation Significantly higher antihyperlipidemic efficacy in Wistar rats	[150]
Epigallocatechin-3-gallate	Chitosan	<200	-	-	Controlled release of encapsulated phytochemical Significantly enhanced antitumor efficacy in 22Rν1 tumor-bearing athymic nude mice	[162]
	Chitosan, polyaspartic acid	102.4 ± 5.6	-	25.0 ± 2.1	Excellent colloidal stability and sustained release of EGCG in gastrointestinal fluids Much better antiatherosclerosis activity in New Zealand white rabbits	[164]
Ursolic acid	Chitosan, poly (lactic acid)	329.3 ± 37.2	27.80 ± 9.4	97.47 ± 1.3	Excellent hemocompatibility and mucoadhesion 3.83-fold improved bioavailability after oral administration in Wistar rats	[171]
	Chitosan, compritol^®^ 888 ATO	103.7 ± 2.8	−24.1 ± 1.6	88.63 ± 2.7	Much higher ex vivo drug absorption by macrophages Much improved in vivo antileishmanial efficacy in Balb/c	[172]
Ferulic acid	Chitosan, phospholipid	123.2 ± 1.11	32 ± 1.28	>90	Almost 2-fold higher intestinal permeation Much higher antioxidant activity in CCl_4_-intoxicated Wistar rats and by restoring the elevated marker enzymes Almost 2.4-fold higher relative bioavailability after oral administration in Wistar rats	[180]
	Chitosan, poly lactic-co-glycolic acid	242 ± 19	32 ± 5	50 ± 4	Good antioxidant activity Comparable cytotoxicity against B16-F10 and HeLa cells Almost 3-fold improved intestinal permeability in Caco-2 monolayers	[181]
10-Hydroxycamptothecin	Chitosan, hyaluronic acid	226.7	-	89.23	Significantly improved intestinal permeation in Caco-2 monolayers Significantly higher cellular uptake and cytotoxicity against Caco-2 cancer cells Excellent antitumor efficacy in Balb/c mice bearing C26 tumors	[185]
	Chitosan, poly lactic-co-glycolic acid	209.9 ± 1.6	6.76 ± 0.27	71.83 ± 2.77	Significantly higher intestinal absorption compared to the free drug due to the inhibition of P-gp efflux transporter Significantly higher cellular uptake and cytotoxicity against Caco-2 cancer cells	[186]
Apocynin	Chitosan, glycerol tristearate	265.3 ± 7.64	40.57 ± 1.0	45.30 ± 2.52	Higuchi’s model-based controlled release of encapsulated apocynin in intestinal fluids 2.52-fold higher relative bioavailability after oral administration in Sprague Dawley rats	[190]
	Chitosan oligosaccharide,	436.2 ± 24.4	38.2 ± 1.47	35.06 ± 1.89	Controlled release of encapsulated apocynin in gastric fluids Much higher antiulcer efficacy on ketoprofen-induced gastric ulcers of Wistar albino rats	[191]
Astaxanthin	Chitosan, poly (ethylene glycol)	122.1 ± 6.4	37.53 ± 2.7	>85	Significantly enhanced intestinal permeability, especially in the jejunum More than 6-fold higher relative bioavailability after oral administration in Sprague Dawley rats	[195]
	Chitosan, caseinate, dextran	91.7–148.8	0.11–0.2	-	Excellent dose-dependent antioxidant activity Excellent biocompatibility and antifibrogenic efficacy in LX-2 cells Significantly reduced the expression level of the TGFβ1-induced fibrogenic gene and COL1A1 protein levels after oral administration in animals	[196]
Berberine	Chitosan, lecithin, dihexadecylphosphate	264 ± 8	29.3 ± 0.5	78.4 ± 0.5	Excellent colloidal stability and controlled release 2.86-fold higher bioavailability after oral administration in New Zealand rabbits	[201]
	Chitosan, fucoidan	187.4 ± 6.2	+7.6 ± 0.5	50.1 ± 2.5	Significantly higher intestinal permeation in Caco-2 cell monolayers Significantly protect the intestinal tight junction barrier function by reducing nitric oxide and inflammatory cytokine levels	[202]
Piperine	Chitosan, glyceryl monostearate	175.3 ± 2.54	−25.24	80.65 ± 1.23	More than 10 times higher ex vivo intestinal permeation 3.76-fold higher bioavailability after oral administration in Wistar rats Significantly strong antidiabetic efficacy in streptozotocin-induced diabetic rats	[207]
Lutein	Chitosan, oleic acid, sodium alginate	125 ± 30	45 ± 5	-	More than 1000-fold improvement in solubility of lutein Strong mucoadhesive property Much higher in vitro intestinal transport in Caco-2 monolayers 128.3% improved bioavailability after oral administration in Wistar rats	[213]
Silymarin	Chitosan, poly(lactic-co-glycolic acid), DSPEPEG2000	286.5 ± 23.8	45.3 ± 8.9	97.05 ± 0.01	Significantly improved intracellular uptake and lipid disposition in HepG2 cells 14.38-fold higher bioavailability after oral administration in Wistar rats Much higher lipid-lowering efficacy in Wistar rats	[219]
Naringenin	Chitosan, sodium alginate	216.44 ± 6	−36 ± 2.7	91.4	Excellent mucoadhesive properties and sustained release of encapsulated naringenin Significantly improved anti-hypoglycemic efficacy in streptozotocin-induced diabetic Wistar rats after oral administration	[226]

## 8. Associated Challenges and Future Outlook

In recent decades, CS-NPs have gained tremendous popularity because of their excellent biodegradable, biocompatible, and non-toxic characteristics. The intestinal absorption and, thereby, oral bioavailability of different phytochemicals loaded in CS-NPs can be enhanced significantly by improving their solubility, and protecting them from pre-systemic metabolism, and enzymatic degradation in the gastrointestinal milieu. However, the toxicological issues of CS-NPs are also a major concern. Although, as a natural polymer, CS is regarded as safe for oral delivery of phytochemicals, its physicochemical properties can be altered completely after chemical functionalization. Therefore, the in vivo toxicity of all CS derivatives should be investigated individually in free form as well as after encapsulation of phytochemicals. Further, pre-clinical investigations are needed to evaluate the bioactivity and safety of CS-based nanocarriers. Another major concern for CS and its derivatives is that their immunological profiles have not been studied thoroughly, and thus far, a complete safety profile is not available. In a review report, Li et al. [40] summarized the different pre-clinical investigations and concluded that CS and CS derivative-based NPs can enhance different immune responses. However, there is no report on toxicological aspects in clinical investigations available. Therefore, further investigation on the clinical immune response is an utmost need in future research. Additionally, few reports claimed that the modification in the deacetylation degree of CS significantly reduces the toxicity. Therefore, extensive pre-clinical and clinical investigations should be conducted in the future to support this claim. The use of different chemicals during the development of CS-based formulations is also a considerable concern. Furthermore, the large-scale production and scalability of different CS-based formulations are other considerable concerns for clinical translation.

In the future, research should be focused on pre-clinical and clinical investigations that are feasible commercially. Although various phytochemical-loaded CS-NPs have been extensively studied both in vitro and in vivo for more than two decades with excellent results, not a single product based on CS-NPs is available in the market for commercial use for the management of chronic diseases. Therefore, phytochemical-loaded CS-NPs should undergo clinical trials, be entered into the market for commercial application, and be economical. In addition, easy and scalable preparation techniques are required for large-scale production of CS-NPs and should come under the “GRAS” category. Most of the preparation techniques for CS-NPs in academic research are not suitable for commercial production due to the use of ingredients and/or techniques that are not viable commercially. Once the suitable techniques for the commercial production of phytochemical-loaded CS-NPs have been established, they need to be extensively investigated using animals and humans to evaluate their bioavailability, bioactivity, and safety after oral administration. After an extensive review of the literature, we expect that further extensive pre-clinical investigation of phytochemical-loaded CS-NPs for oral administration in different animal models will further open the door for clinical trials in humans and will make these products available commercially very soon.

## 9. Conclusions

The present review represents an overview of current strategies on the oral delivery of phytochemical-loaded CS-NPs for improved oral bioavailability and bioactivity. Based on various published investigations, it is evident that CS-NPs could be a potential vehicle for phytochemicals by enhancing their solubility, protecting them from the harsh gastrointestinal environment, avoiding pre-systemic metabolism, improving intestinal permeation, and controlling the release of phytochemicals. Moreover, modification in the structure of CS further improves the phytochemical delivery potential, and more mucoadhesive characteristics, as well as controlled release of phytochemicals, can be achieved. CS-NPs significantly enhance the intestinal permeation, oral bioavailability, and bioactivity of phytochemicals compared to the native phytochemicals after oral administration. In addition, no significant toxicity of phytochemical-loaded CS-NPs has been reported to date. Therefore, CS-NPs can be potential nanoplatforms for improved oral bioavailability and bioactivity of a variety of phytochemicals.

## Figures and Tables

**Table 1:** Physicochemical characteristics of phytochemicals reviewed in this manuscript that are intended for oral administration.

Phytochemicals	Molecular Weight (g/mol)	Aqueous Solubility (mg/mL)	logP Value	pKa Value (Strongest Acidic)	pKa Value (Strongest Basic)	Ref.
Curcumin	368.4	0.00575	4.12	9.06	–4.4	[24]
Quercetin	302.236	0.261	2.16	6.44	–4	[25]
Resveratrol	228.25	0.0688	3.4	8.49	–6.2	[26]
Thymoquinone	164.201	<1	2.55	-	–7.7	[27]
Epigallocatechin-3-gallate	458.372	0.871	3.08	8.73	–3.3	[28]
Ursolic acid	456.7	0.00059	6.58	4.74	–0.84	[29]
Ferulic acid	194.18	0.906	1.67	3.77	–4.9	[30]
10-Hydroxycamptothecin	392.404	0.331	1.69	9.65	3.17	[31]
Apocynin	166.174	3.04	1.62	8.27	–4.9	[32]
Astaxanthin	596.841	0.000667	8.05	13.07	–3.5	[33]
Berberine	336.3612	0.000354	3.6	15	–4.4	[34]
Piperine	285.35	0.149	3.38	12.21	–0.13	[35]
Lutein	568.871	0.000732	8.55	18.22	–0.91	[36]
Silymarin	482.44	0.0926	2.63	7.75	–3	[37]
Naringenin	272.257	0.214	2.84	7.91	–3.9	[38]

**Table 2:** Biological properties of pure chitosan.

Biological Activity	Discussion	Ref.
Antibacterial	CS shows strong antibacterial activity against Gram-positive bacteria (such as *Staphylococcus aureus, Corynebacterium, Staphylococcus epidermidis, Enterococcus faecalis*) as well as Gram-negative bacteria (such as *Escherichia coli, Pseudomonas aeruginosa, Proteus mirabilis, Salmonella enteritidis, Enterobacter aerogenes*), as a result of its polycationic structure.	[39]
Antiviral	The soluble degraded product of CS can effectively inhibit Lucerne mosaic virus and tobacco mosaic virus.	[40,41]
Antifungal	CS derivatives with a large charge density can effectively inhibit different fungi such as *Candida albicans* and *Candida parapsilosis.*	[39,42]
Wound healing	Pure CS is widely used as a wound dressing material due to its excellent wound healing activity.	[39,43]
Anticancer	Low-molecular weight CS and chito-olegosaccharide could significantly reduce tumor growth.	[44,45,46]
Anti-inflammatory	CS shows anti-inflammatory activity by inhibiting the production of cytokines and keratinocytes.	[47]
Immunostimulatory	CS and CS derivatives effectively activate antigen-presenting cells by different mechanisms and induce cytokine stimulation to produce an effective immune response.	[48]

**Table 3:** Advantages and limitations of CS-NPs over other NP-based systems for oral delivery of phytochemicals [71].

Advantages of CS-NPs	Limitations of CS-NPs
Negligible toxicity and safe for oral administration	Low solubility in neutral and alkaline pH
Biodegradable and biocompatible	Method of development is complex and mainly depends on the therapeutic molecules to be encapsulated
Excellent stability in the harsh gastrointestinal milieu	Less mechanical resistance
Excellent mucoadhesive characteristics	Electrospinning for pure CS is difficult
Controlled release of encapsulated molecules	Large pore size
Site-specific targeted delivery of therapeutic molecules	Covalent cross-linking can modulate the intrinsic characteristic of pure CS
Improves mean residence time in the GIT	
Improves intestinal permeation by opening of TJs	
Inhibits P-gp efflux transporter	

## Data Availability

Not applicable.
